# The Physician’s Visiting List

**Published:** 1877-10

**Authors:** 


### THE PHYSICIANS' VISITING LIST.

This invaluable little work for 1878 by Lindsay & Blakiston, is just from the press. It is most complete and perfect in its arrangement, and answers every requirment of the physician in his direction. It may be used by the dentist as well, and indeed it is as good an engagement book for the dentist as we have seen. It is neat and compact, and contains besides its engagement pages, much valuable space and matter. It can be obtained at the Dental Depots and Book Stores.

